# A photogrammetric image-point dataset for the semantic segmentation of heritage buildings

**DOI:** 10.1016/j.dib.2025.111661

**Published:** 2025-05-14

**Authors:** Eugenio Pellis, Andrea Masiero, Michele Betti, Grazia Tucci, Pierre Grussenmeyer

**Affiliations:** a Department of Civil and Environmental Engineering (DICEA), University of Florence via di S. Marta 3, Florence 50139, Italy; b Interdepartmental Research Center of Geomatics (CIRGEO), TESAF Department, University of Padova, viale dell’Università 16, Padova 35020, Italy; c INSA Strasbourg, CNRS, ICube Laboratory UMR 7357, Université de Strasbourg, Boulevard de la Victoire Strasbourg 67000 France

**Keywords:** Cultural heritage, Images, 3D point clouds, Terrestrial photogrammetry, Semantic segmentation

## Abstract

In this article, we present a new dataset designed for the semantic segmentation of images and point clouds of historic buildings, aimed at automating and accelerating the 3D modeling process in the scan-to-BIM context. The dataset includes five historic buildings and provides two types of data: images obtained from photogrammetric surveys and the corresponding georeferenced point clouds. Both datasets are accompanied by ground truth, identifying 10 classes representative of the main construction elements. Additionally, the dataset includes the intrinsic and extrinsic camera parameters and the transformation matrix to align the point clouds with the camera reference system. The annotation of the point clouds was performed manually, while the image annotation was generated through a projection process based on the labels assigned to the point clouds.

Specifications table

**Table:** 

Subject	Computer Sciences
Specific subject area	Supporting the creation of three-dimensional informative models of historical buildings, throughout the 3D semantic segmentation of the standard BIM-based structural elements.
Type of data	Images (.png), Point clouds (.txt), Text File (.txt).
Data collection	The photogrammetric images were acquired using close range photogrammetry technique with high image overlap in order to obtain the 3D reconstruction of each buildings. Two digital single-lens reflex (DSLR) camera was used, a Nikon D60 and a Nikon D80, both with 10.2 MP, provided with a 23.6 mm x 15.8 mm Nikon DX format RGB CCD sensor, 1.5 x FOV crop, with a maximum resolution of 3,872×2,592 pixels. The camera was equipped with a zoom lens AF-P DX Nikkor 18-55 mm f/3.5-5.6G. The images were acquired in the .JPEG format. All the images were acquired with a focal length of 18 mm, ISO-200, and a fixed aperture of f/14. The related photogrammetric point clouds have been created with Agisoft Metashape.
Data source location	Institution: Department of Civil and Environmental Engineering (DICEA) City/Town/Region: Florence, Tuscany County: Italy
Data accessibility	Repository name: Zenodo Data identification number: (doi:10.5281/zenodo.14626611) Direct URL to data: (10.5281/zenodo.14626611)
Related research article	‘none’

## 1 Value of the Data

• The dataset consists of five heritage buildings. For each scene, two types of data are available: photogrammetric images with corresponding intrinsic and extrinsic parameters, and the related point cloud. Annotated ground truth is provided for both types of data.
• The dataset is particularly suited for training, validating, and testing machine learning models, and it is mainly focused to support automation in three-dimensional and informative model generation via semantic segmentation.
• These multi-source data can be valuable in several ways: implementing point-based and multi-view-based approaches, comparing their accuracy on the same data source, and developing new hybrid networks that leverage both images and point clouds.
• The dataset adheres to the classification guidelines of ARCHdataset, enabling potential integration to enhance its capabilities and to support the development of more general classification models.

## 2 Background

Heritage Building Information Modelling (H-BIM) has received increasing attention in recent years due to a heightened focus on the protection, conservation, and restoration of historical buildings throughout digital technology [1,2]. The transformation of 3D point clouds into comprehensive three-dimensional models is a complex task that demands significant manual effort from skilled operators and is hampered by the absence of standardized procedures and methods to expedite the process [3]. Developing an effective semantic segmentation technique, which involves categorizing a 3D point cloud into meaningful classes based on scene interpretation, is essential for automating and speeding up this workflow [4]. Various techniques have been effectively utilized for 3D point cloud semantic segmentation [5]. In recent years, machine learning (ML) and deep learning (DL) methods have achieved notable success in numerous segmentation applications. There are several works dealing with ML and Cultural Heritage (CH) sector [[6], [7], [8]]. However, their application still remains limited, and the results published thus far are not entirely satisfactory. One contributing factor to these underwhelming results is the limited availability of dedicated benchmarks specifically designed for segmentation tasks in the heritage context. To face these challenges, this paper introduces a new image-point cloud dataset designed for the semantic segmentation of heritage buildings.

## 3 Data Description

**Fig. 1:**
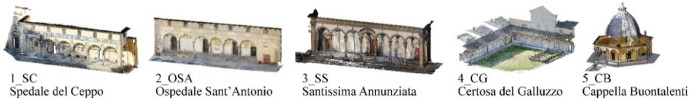
The five building of the dataset.

The dataset consists of five heritage building scenes (Fig. 1). These five buildings are located in Tuscany, Italy, and they were constructed in various historical periods, showcasing diverse architectural styles. Despite these differences, they share common architectural features typical of the Florentine Renaissance style, such as facades with loggias, classical orders or similar design proportions. For each scene, two types of data are available: photogrammetric images with corresponding intrinsic and extrinsic parameters, and the related point cloud. Annotated ground truth is provided for both types of data.

**Fig. 2:**
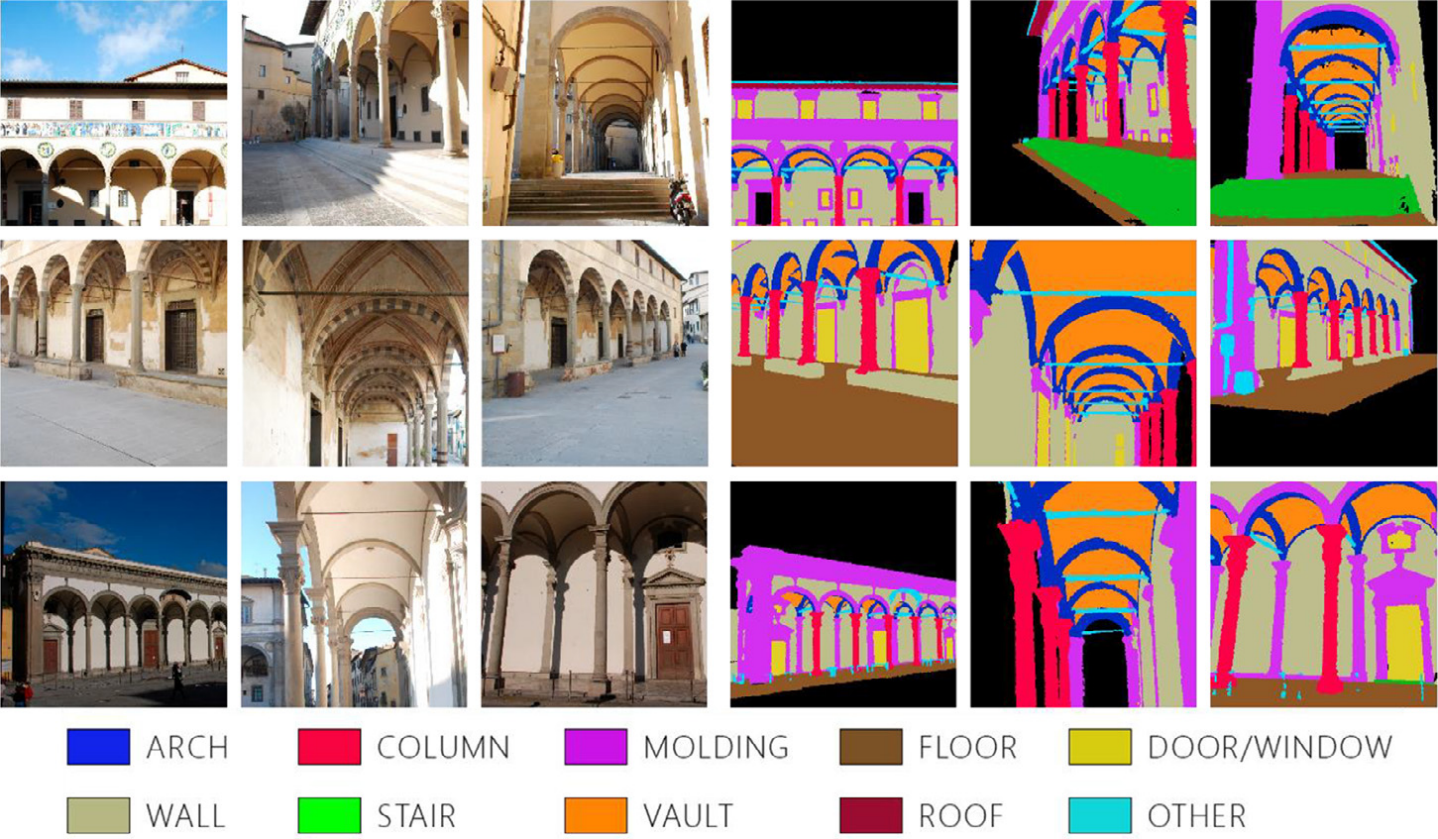
Some images of the dataset with the corresponding ground truth.

Given that the primary goal of the dataset is to support and facilitate 3D model generation in a BIM environment, it is crucial to select the segmentation output in accordance with the standards and element categories of the leading BIM-based or object-oriented software. Hence, the segmentation categories in this dataset adhere to the standards and guidelines established by the ARCHdataset [9]. As a result, the point cloud scenes are segmented into 10 classes. The classes and the related indexes are the following: *‘arch’* : *0, ‘column’* : *1, ‘moulding’* : *2, ‘floor’* : *3, ‘door/window’* : *4, ‘wall’* : *5, ‘stair’* : *6, ‘vault’* : *7, ‘roof’* : *8, ‘other’* : *9.* Unlike point clouds, images always contain a background, necessitating the introduction of a new class that includes all pixels not classified under the existing categories. This class is conventionally named *’background’* and it is labelled with the index *10*. Additionally, the *‘other’* class encompasses elements not covered by the predefined classes but are either part of or adjacent to the building. Some images of the dataset with the corresponding ground truth are show in Fig. 2.

We organized the collected data into five main folders, each corresponding to a specific building. These folders are labelled sequentially from 1 to 5, along with an abbreviation for each building (e.g., 1_SC). Inside each folder, there are two subfolders: *‘1_Images’* and *‘2_PointCloud’*.

The *'1_Images'* subfolder contains three further subfolders:

• *1_RGB*: This subfolder contains all the RGB photogrammetric images in *.png* format, named using the building abbreviation followed by an incremental number (e.g., 1_SC_001).
• *2_labels*: This subfolder holds the corresponding ground truth maps, also in *.png* format, following the same naming convention.
• *3_camera_info*: This subfolder includes two files. The first, *‘1_interior_parameters.txt’*, contains the intrinsic camera matrix *‘K’*, radial distortion coefficients *‘ks’*, and tangential distortion coefficients *‘ps’*. The second file, *‘2_exterior_parameters.txt’*, provides the exterior camera parameters for each image, listed in the following sequence: PhotoID, X, Y, Z, Omega, Phi, Kappa, r11, r12, r13, r21, r22, r23, r31, r32, r33, orientation. The ‘*orientation’* parameter indicates whether the image reflects the correct building orientation. A value of *0* means the image is correct, while *1* indicates it needs rotation. This ensures neural networks are trained with properly oriented images.

The second main subfolder, *'2_PointCloud'*, contains two files:

• *1_point_cloud*: This file represents the scaled and georeferenced point cloud in .txt format, with data organized as (X, Y, Z, R, G, B, label, Nx, Ny, Nz).
• *2_transformation_matrix*: This file contains the matrix used to transform the point cloud and align it with the coordinate system of the photogrammetric images.

**Fig. 3:**
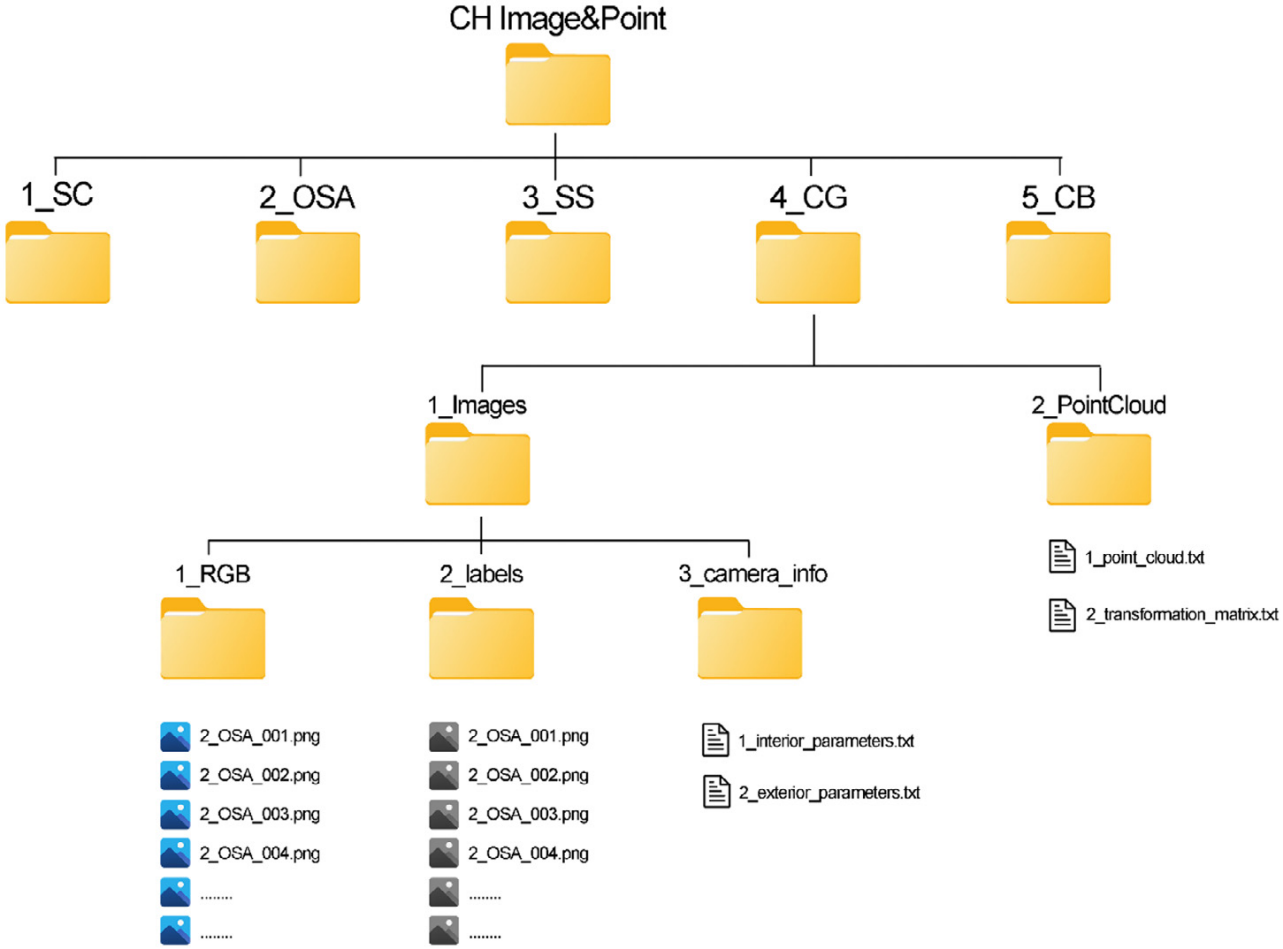
The hierarchical structure of the data.

Fig. 3 illustrates the hierarchical structure of the dataset, which is hosted on Zenodo.

## 4 Experimental Design, Materials and Methods

This section covers the methods used for data collection, pre-processing, and the creation of ground truth segmentation. Each subsection includes a brief explanation of the techniques and tools employed, accompanied by relevant illustrations.

### 4.1 Data Collection

The terrestrial laser scanner (TLS) data, collected by the GECO laboratory (Geomatics and Conservation group of the Department of Civil and Environmental Engineering, University of Florence) were already available for all the mentioned buildings. These point clouds are expressed in a local metric Cartesian coordinate system, with the z-axis coinciding with the vertical direction. Based on the specific building, the photogrammetric survey was conducted using two digital single-lens reflex (DSLR) cameras: a Nikon D60 and a Nikon D80. Both cameras have a resolution of 10.2 megapixels and are equipped with a 23.6 mm x 15.8 mm Nikon DX format RGB CCD sensor, featuring a 1.5x FOV crop factor and a maximum resolution of 3,872×2,592 pixels. The cameras were fitted with AF-P DX Nikkor 18-55 mm f/3.5-5.6G zoom lenses. Images were captured in JPEG format. All shots were taken at a focal length of 18 mm, ISO-200, and with an aperture no smaller than f/14. This ensured a good depth of field, optimized the photogrammetric process, and enhanced the number of usable pixels for point cloud reconstruction. Table 1 provides further details on each acquisition.

**Table 1:** Details on data acquisition.

Building	TLS cloud	Camera	N° of photo	Resolution
1_SC	yes	Nikon D60	**748**	3872×2592
2_OSA	yes	Nikon D60	**755**	3872×2592
3_SS	yes	Nikon D80	**473**	3872×2592
4_CG	yes	Nikon D60	**1102**	3872×2592
5_CB	yes	Nikon D80	**166**	3872×2592

### 4.2 Data Pre-Processing

The collected images were pre-processed firstly to create the 3D scene, and secondly to set them up for the annotation phase. For each scene the processing operations followed several steps.

*Cloud Generation.* The initial processing step involved constructing the point cloud from the images. For this purpose, we used Agisoft Metashape™. This process produces an RGB point cloud along with the associated intrinsic and extrinsic camera parameters, which are calculated during the camera alignment phase.

*Cloud alignment*. The photogrammetric surveys, lacking targets and reference points, produced a dimensionless, non-georeferenced point cloud. Hence, the point cloud was scaled and placed in a local metric reference system by aligning it with the available TLS point cloud through a two-step process: a manual rough alignment using manually picked reference points, followed by refinement with the Iterative Closest Point (ICP) algorithm. The corresponding transformation matrix is provided along with the data. This matrix allows the photogrammetric point cloud to be transformed back into the initial reference system, which coincides with that of the photogrammetric images provided in the file “*2_exterior_parameters*”.

*Cleaning/Denoising.* These operations were essential to remove unwanted parts of the scene and improve point cloud accuracy. This was done manually by deleting unwanted points or semi-automatically using features selection like colour, suited for sky and vegetation or geometry (e.g., planarity, distance, altitude).

*Subsampling.* This operation was used to reduce point density and homogenize the scenes while maintaining detail. A random subsampling with a minimum distance of 0.01 m was chosen to balance file size, computational cost, and detail accuracy.

### 4.3 Data Annotation

**Fig. 4:**
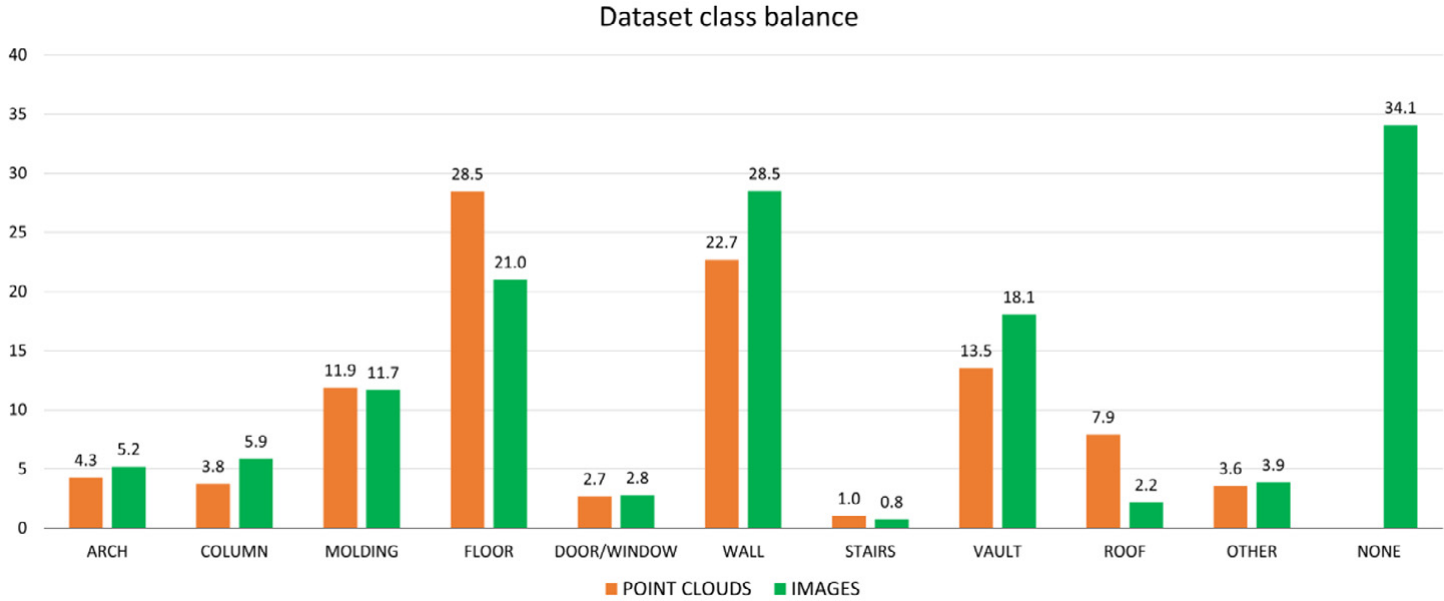
Class balancing for the point clouds (orange), and for the images (green).

The data annotation process was carried out in two main stages. In the first stage, the point cloud was manually annotated by selecting points of interest using bounding boxes or leveraging geometric properties such as distance, planarity, altitude, and symmetry. The second stage involved image annotation, achieved through an automatic projection process. The projection procedure requires as input the segmented 3D scene and the internal and external camera parameters obtained from the photogrammetric workflow. The output consists of all the corresponding segmented photogrammetric images, where the ground-truth labels are derived from the annotations of the 3D scene. The projection of 3D points onto the image plane is performed while ensuring visibility criteria and the absence of obstructions. To mitigate gaps caused by low point density or areas with missing points, several adaptive parameters are employed to expand the labelled area based on the distance from the camera. Finally, two corrective functions are applied to eliminate misclassified pixels and reduce unlabelled regions, thereby improving the accuracy of the final segmentation. The procedure has demonstrated a global accuracy of 97 %, as reported in [10], where further details on the methodology are provided. This approach enables the simultaneous labelling of a large number of images from a single point cloud, significantly reducing the time and resources required while ensuring perfect consistency between the labels of the point cloud and those of the corresponding images. The percentages of the various classes relative to the total points for the point clouds or the total pixels for the images are shown in the graph below (Fig. 4).

## Limitations

The proposed dataset still contains some limitations. Regarding the size, the image dataset currently consists of 3,244 images. Although this substantial amount of data is sufficient for training a neural network, it is important to note that the images depict thousands of different views of the same buildings from various distances, angles, and perspectives. Consequently, the dataset primarily represents a narrow range of building types. The dataset reveals a significant class imbalance, with a notable predominance of the *“floor”* and *“wall”* classes and a limited representation of the *“stair”, “door/window”*, and *“other”* classes. The image dataset also indicates a predominance of pixels labelled as *“background”* in the image dataset. Unlike point clouds, this class is inherently present and includes a broad range of element types, some of which may resemble building elements. Concerning the data quality, as detailed in the previous paragraph, the ground truth for the images was created using a projection method based on a manually segmented point cloud. While this procedure works correctly, some limitations of this method should be noted. Firstly, due to the discontinuous nature of the point cloud and the presence of low-density regions, some ground truth images have “empty” areas. These areas are automatically classified as *"background"*. Secondly, obstructions and moving objects in the images were not captured in the point cloud, leading to their absence in the image classifications. Thirdly, the point-based nature of the cloud causes the edges of objects in the images to be pixelated and not perfectly defined.

## Ethics Statement

The authors have read and follow the ethical requirements for publication in Data in Brief and confirming that the current work does not involve human subjects, animal experiments, or any data collected from social media platforms.

## Declaration of Generative AI-assisted Technology

During the preparation of this work the authors used AI-assisted technologies in order to improve the readability and language of the manuscript. After using this tool/service, the authors reviewed and edited the content as needed and take full responsibility for the content of the published article.

## CRediT authorship contribution statement

**Eugenio Pellis:** Methodology, Investigation, Validation, Writing – original draft, Data curation. **Andrea Masiero:** Methodology, Supervision, Writing – review & editing. **Michele Betti:** Supervision, Writing – review & editing. **Grazia Tucci:** Supervision, Resources. **Pierre Grussenmeyer:** Supervision.

## Data Availability

ZenodoImages&PointClouds Cultural Heritage Dataset (Original data)
